# Trend of Bloody Diarrhea in Kerman Province, Iran: A Time Series Study From 2013 to 2023

**DOI:** 10.1155/cjid/8516211

**Published:** 2026-04-10

**Authors:** Zahra Jaafari, Andishe Hamedi, Zahra Abdolahinia, Saeid Sohbati, Ali Esmaeilpour, Mozhgan Seif

**Affiliations:** ^1^ Student Research Committee, Department of Epidemiology, School of Health, Shiraz University of Medical Sciences, Shiraz, Iran, sums.ac.ir; ^2^ Department of Biostatistics and Epidemiology, Kerman University of Medical Sciences, Kerman, Iran, kmu.ac.ir; ^3^ Department of Prevention and Control of Communicable Diseases, Kerman University of Medical Sciences, Kerman, Iran, kmu.ac.ir; ^4^ Department of Epidemiology, School of Health, Shiraz University of Medical Sciences, Shiraz, Iran, sums.ac.ir

**Keywords:** bloody diarrhea, epidemiology, Iran, Kerman

## Abstract

**Background:**

Bloody diarrhea is a significant infectious disease of the gastrointestinal tract that poses a serious public health concern.

**Objective:**

This study aims to elucidate the epidemiological profile of bloody diarrhea in Kerman Province, Iran, highlighting the necessity for targeted public health interventions.

**Methods:**

This longitudinal study analyzed data from 3111 patients diagnosed with bloody diarrhea between 2013 and 2023 in Kerman Province, Iran. The Generalized Estimating Equations (GEE) method was employed to identify predictors of bloody diarrhea incidence. To investigate trends over time, the Box–Jenkins approach was utilized to model the autoregressive integrated moving average (ARIMA) time series (*p*, *d*, *q*).

**Results:**

The findings revealed a significantly higher incidence of bloody diarrhea among men (55.5%), children under 6 years old (48.0%), and individuals residing in urban areas (76.7%). The clinical assessment indicated that a substantial majority of patients were hospitalized (72.1%). Laboratory analyses identified *Shigella* species as the most prevalent pathogen (56.0%). The GEE analysis demonstrated that the risk of developing bloody diarrhea was particularly elevated in children under six and urban residents. The ARIMA model indicated seasonal components in the incidence of bloody diarrhea, with a mild decreasing trend observed over the study period.

**Conclusions:**

Given the clinical significance of this disease and its impact on vulnerable populations, particularly children, we advocate for enhanced community education and the strict implementation of health protocols to improve disease management and prevention.

## 1. Introduction

Diarrhea is the third leading cause of death in children under five years of age, accounting for 1.7 billion cases of childhood diarrheal disease and 443,832 deaths annually in 2024, according to the World Health Organization (WHO). Diarrhea can last for several days and leave the body without the water and minerals it needs to survive. [1]. Any episode of diarrhea in which there is visible blood in loose or watery stools is clinically diagnosed as bloody diarrhea or dysentery [2]. Bloody diarrhea is a characteristic of intestinal infections, mainly caused by invasive bacteria that can be spread through direct fecal‐oral contamination through the feces of an infected person [3].

In the past, for most people, severe dehydration and fluid loss were the leading cause of death from diarrhea. Now, other causes, such as bacterial infections, are likely to account for an increasing share of all diarrhea‐related deaths [1]. Bacterial dysentery can be caused by four species of *Shigella* (*Shigella dysenteriae*, *Shigella flexneri*, *Shigella boydii*, and *Shigella sonnei*) [4]. *Shigella* dysentery is an acute bacterial disease that is the most common cause of bloody diarrhea. The disease is characterized by diarrhea accompanied by fever, nausea, vomiting, and muscle cramps, which is called shigellosis. The blood and mucus in the diarrhea are the result of the invasion of microorganisms and the destruction of the cells lining the small and large intestines [5–7]. Cultural and geographical factors and possibly antibacterial drug use patterns are influential in *Shigella* serotypes and drug resistance [8–10].

In addition to bacterial pathogens, several nonbacterial etiologic agents play an important role in the development of bloody diarrhea. Amoebiasis caused by *Entamoeba* histolytica is one of the most common parasitic causes of dysentery, particularly in developing countries, and is associated with invasive intestinal disease and bloody stools [11]. Certain pathotypes of *Escherichia coli*, especially enterohemorrhagic *E. coli* (EHEC), such as *E. coli* O157:H7, can also cause bloody diarrhea through the production of Shiga toxins, which damage the intestinal epithelium and may lead to severe complications, including hemolytic uremic syndrome [12]. In addition, other parasitic agents such as *Balantidium coli* and *Schistosoma species*, as well as viral pathogens including cytomegalovirus (particularly in immunocompromised individuals), have been reported as less common but significant causes of bloody diarrhea [13]. Therefore, accurate identification of the etiologic agents of bloody diarrhea is essential for appropriate treatment, surveillance, and effective public health intervention.

Conventional antibiotic treatments are generally used for infectious bacteria, which play a vital role in reducing the prevalence and mortality of these infections, but repeated and inappropriate use of an antibiotic leads to the emergence of new resistant strains [14]. Therefore, understanding the conditions of disease occurrence at different times can help in control, prediction, surveillance, program review, policy analysis, and its etiology. Examining the trend of indicators and considering their changes helps health service providers to evaluate the efficiency of health systems at different times and determine to what extent the implementation of programs using different facilities has been successful in achieving their goals. In addition, identifying changes in the trend of disease occurrence can be of great importance in evaluating the efficiency of health control programs, the performance of health care personnel, and decision‐making in health programs. Given the importance of the issue, we conducted this study with the aim of examining the trend of bloody diarrhea in Kerman Province of Iran, between 2013 and 2023.

## 2. Materials and Methods

### 2.1. Design and Participants

This longitudinal study was conducted in Kerman Province. Kerman Province is located in southern Iran, and its population, according to the Statistics Center of Iran, was 3,164,718. Kerman is the largest province in Iran, covering more than 11% of Iran’s area, with an area of about 183,193 square kilometers [15]. This study analyzed data from 3111 cases of bloody diarrhea recorded in the Kerman University of Medical Sciences water and food‐borne disease surveillance system. Bloody diarrhea was defined as the presence of visible blood accompanied by loose, watery stools [16]. The management process for bloody diarrhea within the health system includes the following steps:

After completing personal and epidemiological information forms, patients provide stool samples to laboratories with informed consent. Stool samples are processed and tested within a maximum of 6 h. Following a standard protocol, trained technicians culture all samples in media designed for *Shigella*, *Salmonella*, the bacterium *Vibrio cholerae*, and *Campylobacter* bacteria. This study focused solely on identifying bacterial pathogens, and no parasitological examination or microscopic examination of feces was performed to identify nonbacterial agents. The culture performed was performed without identifying the speciation and, therefore, these cases may include both symbiotic and pathogenic strains.

In this study, microscopic evaluation of red and white blood cells (WBCs) in stool samples was performed using standardized and reproducible methods. Fresh stool samples (collected within 30 min of defecation) were obtained from blood‐stained or mucoid portions, and in cases of delay, samples were preserved in 10% formalin. For the assessment of red blood cells (RBCs), the wet mount method without staining was employed. A drop of stool suspension was placed on a glass slide and immediately examined under a microscope at 10× and 40× magnifications. RBCs were observed as round, homogeneous, and anucleate cells, with their presence indicating lower gastrointestinal bleeding. For WBC evaluation, methylene blue and Wright/Giemsa stains were used. Methylene blue staining selectively colored WBC nuclei dark blue while preserving cellular morphology, while Wright/Giemsa staining allowed differentiation of leukocyte types. The presence of neutrophils indicated inflammatory diarrhea (e.g., bacterial infections), whereas eosinophils were associated with parasitic infections or inflammatory bowel diseases. These methods ensured high accuracy and clear diagnostic standards for the analysis of clinical samples.

This is a population‐based study that considered all patients diagnosed with bloody diarrhea in Kerman Province between 2013 and 2023, identified through a census method utilizing epidemiological survey forms (personal characteristics and diagnostic test reports). Patients (4 cases) with incomplete file information (no reporting of the year of incidence) were excluded from the study. Required information was extracted using a checklist based on predetermined variables. Demographic characteristics (age, gender, place of residence, and clinical status) and laboratory test results were obtained by reviewing patient files. In this study, patients were classified into two clinical status categories: outpatient and inpatient. Outpatient cases were those who were managed at primary healthcare centers or outpatient clinics without hospital admission. Inpatient cases were defined as patients admitted to a hospital for at least 24 h due to the severity of illness or need for inpatient care. The denominator for both categories was the total number of reported bloody diarrhea cases during the study period.

### 2.2. Statistical Analysis

In this study, descriptive statistics were employed to summarize the data, with means ± standard deviations (SD) used for quantitative variables and frequencies for qualitative variables.

To estimate the number of cases of bloody diarrhea while accounting for independent variables, we employed Generalized Estimating Equations (GEE). We used Poisson GEE with a log link to model monthly city‐level incidence counts, offset by city‐month population to estimate incidence rates. Observations were clustered within cities, with correlation over time addressed via a working AR [1] correlation structure. Time was included as a covariate to capture long‐term trends. Robust sandwich standard errors were used to obtain valid inference under potential correlation structure misspecification. We compared alternative correlation structures via the quasi‐information criterion and performed sensitivity analyses using negative‐binomial GEE to address potential overdispersion. The results were robust to these specification choices, supporting the validity of our population‐averaged inferences about factors influencing the incidence rate. The GEE model is advantageous in longitudinal studies as it can accommodate unequal numbers of observations from different individuals.

Given the potential seasonal trends in the data, ordinary regression methods were deemed inappropriate due to the correlation of observations within each season. Thus, we utilized GEE to estimate regression coefficients while considering the correlation structure of the data.

The statistical analysis was performed using SPSS version 26, with a significance level set at (*p* < 0.05).

To examine trends in the data, we implemented the Box–Jenkins approach to model the autoregressive integrated moving average (ARIMA) time series, denoted as ARIMA(*p*, *d*, *q*). The province‐level time series comprises monthly counts of disease incidence from January 2013 to December 2023. The objective of this analysis is short‐term forecasting of total cases at the provincial level to inform public health planning. The parameters of the ARIMA model are defined as follows:

• (*p*): the order of the autoregressive part
• (*d*): the degree of differencing
• (*q*): the order of the moving average part.

The ARIMA model can be expressed mathematically as follows:

(1) Yt=φ1Yt−1+φ2Yt−2+…+φpYt−p+θ1εt−1+θ2εt−2+…+θqεt−q+εt,

where (*Y*_*t*_) is the time series at time (*t*), (*φ*) are the autoregressive coefficients, (*θ*) are the moving average coefficients, and (*ε*) is white noise.

In addition, seasonal ARIMA models are typically denoted as (m) ARIMA(*p*, *d*, *q*) (*P*, *D*, *Q*), where (m) is the number of periods in each season, and the capital letters (*P*), (*D*), and (*Q*) represent the seasonal autoregressive, differencing, and moving average terms, respectively. Given the seasonal nature of our data, we utilized a general multiplicative seasonal ARIMA model of the form:

(2) 12 ARIMA p, d, qP, D, Q.

To determine the order of the moving average (MA) and autoregression (AR) terms in the ARIMA model, we employed autocorrelation function (ACF) and partial ACF (PACF) plots. Model parameter estimation was performed using the conditional least squares method.

We conducted diagnostic analyses, including residual analysis and the Akaike Information Criterion (AIC), to compare the goodness of fit among the ARIMA models. We examined residuals for white noise behavior, checked for autocorrelation, and evaluated model adequacy; we utilized the Ljung–Box test to assess prediction accuracy. Finally, the fitted ARIMA model was applied to predict the short‐term occurrence of bloody diarrhea cases for the years 2024–2033, with data analyzed using R software.

## 3. Results and Discussion

In this study, 3111 individuals with bloody diarrhea were examined. The mean age of those affected was 16.67 years, with a standard deviation of 20.24. The findings revealed a higher incidence of bloody diarrhea among men (55.5%), children under 6 years old (48.0%), and individuals residing in urban areas (76.7%). Clinical assessments indicated that a substantial majority of patients were hospitalized (72.1%) (Table 1). Laboratory results, as shown in Table 2, identified *Shigella* species as the most prevalent pathogen, accounting for 56.0% of cases.

**TABLE 1:** Demographic characteristics of patients with bloody diarrhea in Kerman province, Iran, during 2013–2023 (*N* = 3111).

Variable	Categories	Frequency (%)
Gender	Male	1727 (55.5)
	Female	1384 (44.5)

Age groups (year)	Under 6	1492 (48.0)
	7–29	920 (29.6)
	30–59	502 (16.1)
	≥ 60	197 (6.3)

Place of residence	Urban area	2386 (76.7)
	Rural area	725 (23.3)

Clinical status	Inpatient	2242 (72.1)
	Outpatient	869 (27.9)

**TABLE 2:** Laboratory factors of patients with bloody diarrhea in Kerman province, Iran, during 2013–2023 (*N* = 3111).

Type of microbe observed	Positive, *N* (%)
*Shigella*	249 (56.0)
*Salmonella*	20 (4.5)
*E. coli*	34 (7.6)
Other	5 (1.1)
Unspecified	137 (30.8)

*Note:* Table information is based on the results of 445 patients who had positive culture results (the remaining subjects had no stool culture). *E. coli* isolates were not characterized for pathogenicity; results may include both commensal and pathogenic strains. *Shigella* was identified to genus level without further speciation.

**FIGURE 1:**
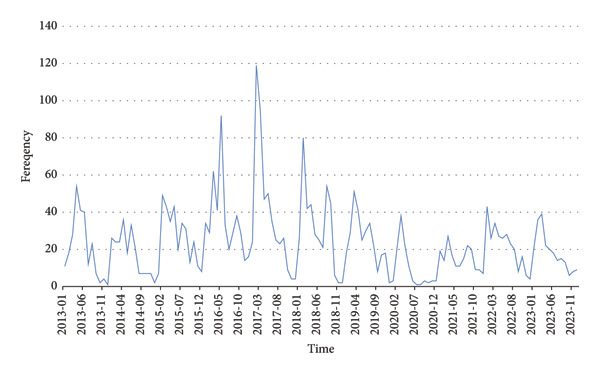
Trend in bloody diarrhea incidence in Kerman province, Iran, during 2013–2023 (*N* = 3111).

**FIGURE 2:**
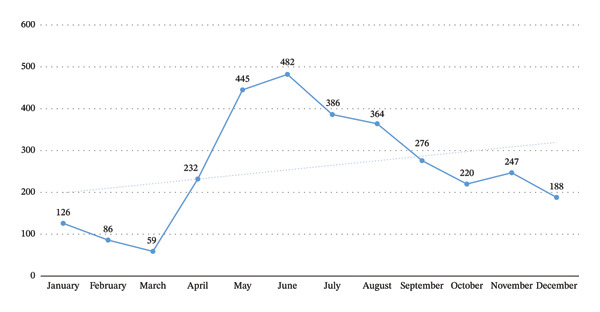
Seasonal changes in the incidence of bloody diarrhea by month in Kerman province, Iran, during 2013–2023 (*N* = 3111).

GEE analysis demonstrated that the risk of developing bloody diarrhea was particularly elevated in children under six and urban residents. Additionally, the risk of hospitalization was significantly higher among these patients (Table 3). According to the ARIMA model, the incidence of bloody diarrhea in Kerman Province decreased from 2013 to 2023 (Figure 1). Figure 2 illustrates seasonal variations, with the highest number of cases occurring in June and May, respectively.

**TABLE 3:** Rate ratio of factors associated with bloody diarrhea in Kerman province, Iran, during 2013–2023 (*N* = 3111).

Variable	Categories	Frequency (%)	Rate ratio (95% CI)	*p* value
Gender	Male	1727 (55.5)	1.24 (0.92–1.69)	0.15
	Female	1384 (44.5)	Reference	—

Age groups (year)	Under 6	1492 (48.0)	7.57 (4.86–11.79)	< 0.001
	7–29	920 (29.6)	4.67 (2.94–7.40)	< 0.001
	30–59	502 (16.1)	2.54 (1.51–4.27)	0.001
	≥ 60	197 (6.3)	Reference	—

Place of residence	Urban area	2386 (76.7)	3.29 (2.35–4.60)	< 0.001
	Rural area	725 (23.3)	Reference	—

Clinical status	Inpatient	2242 (72.1)	2.58 (1.56–4.25)	< 0.001
	Outpatient	869 (27.9)	Reference	—

Figure 3 presents the use of the ACF and the PACF to analyze random, fixed, and seasonal effects on time series data. Based on the distribution characteristics, the ARIMA (1, 1, 1) (1, 0, 0) 12 model was determined to be the best fit for the data, as it had the lowest AIC value. A comparison of the tested models is shown in Table 4. The Ljung–Box test for model validation indicated that the current model exhibited no autocorrelation and demonstrated relatively good accuracy (*Q* = 5159, *p* > 0.05).

FIGURE 3Autocorrelation function (ACF) and partial autocorrelation function (PACF).

**Figure:**
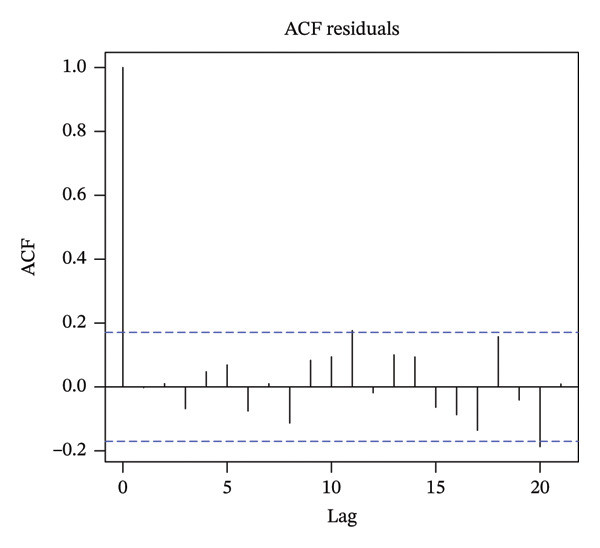
(a)

**Figure:**
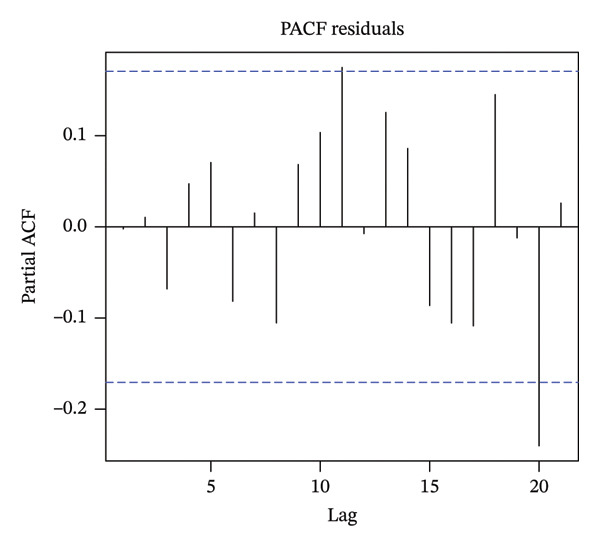
(b)

**TABLE 4:** Comparison of tested models.

Model	AIC
ARIMA (1, 1, 0) (1, 0, 0) [12]	1120.765
ARIMA (0, 1, 1) (0, 0, 1) [12]	1115.498
ARIMA (0, 1, 1) (0, 0, 2) [12]	1114.11
ARIMA (0, 1, 0) (0, 0, 2) [12]	1130.434
ARIMA (0, 1, 2) (0, 0, 2) [12]	1100.454
ARIMA (0, 1, 2) (0, 0, 1) [12]	1099.587
ARIMA (0, 1, 2) (1, 0, 1) [12]	1095.107
ARIMA (0, 1, 2) (1, 0, 0) [12]	1096.143
ARIMA (0, 1, 2) (2, 0, 0) [12]	1097.31
ARIMA (1, 1, 1) (0, 0, 1) [12]	1094.80
ARIMA (1, 1, 1) (1, 0, 0) [12]	1091.65

**FIGURE 4:**
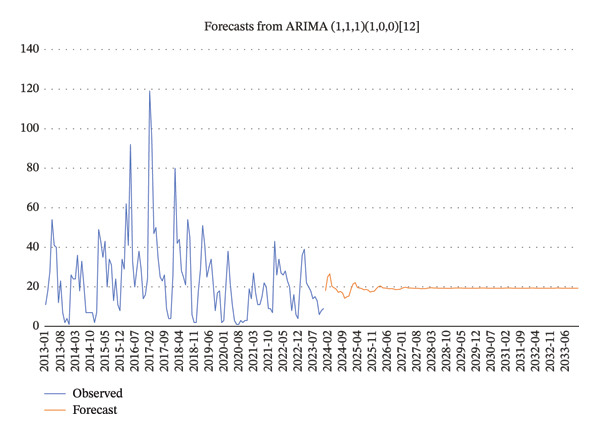
Prediction incidence of bloody diarrhea in Kerman province, Iran, during 2024–2033.

Predicted data for 2024 to 2033 show a decreasing trend in the incidence of bloody diarrhea cases. However, this decline is gradual, suggesting that the predicted incidence rates are likely to stabilize at a certain level in the coming decade (Figure 4).

In this study, we showed that more than half of bloody diarrhea patients were male, and the risk of developing bloody diarrhea was particularly elevated in children under six and urban residents. Similar to our findings, national data from nine countries using the meta‐analytic method showed that *Shigella* infection is more common in males than females, particularly in children, which may be related to higher exposure to outdoor environments and greater contact with contaminated sources [17]. Also, the risk of hospitalization was higher among these patients. Furthermore, more than half of the cases were associated with the *Shigella* pathogen, and the highest number of cases occurred in June and May, respectively. A gradual decline in bloody diarrhea cases was shown over the years, likely stabilizing at a certain level over the coming decade.

Similar to our result, a study in India had more male patients than female [18]; however, contrary to our findings, bloody diarrhea in the Indian study was more common in rural areas than in urban areas [18]. Differences in sanitation infrastructure and water supply systems, population density, environmental health, migration and demographic changes, and access to health services in each country could affect the results. Urban areas, often characterized by higher population density and increased person‐to‐person contact, may facilitate the transmission of pathogens like *Shigella*. Conversely, rural areas may face inadequate sanitation and limited healthcare access, which can also elevate disease risk [19].

As our results revealed, younger children, especially infants and children under 6 years of age, are likely to be at higher risk of hospitalization for bloody diarrhea due to their weaker immune systems, faster rate of dehydration, and the type of food and diet. Infants and young children can less compensate for fluid loss, making early recognition and management of diarrhea crucial [20, 21]. It seems that educating parents about the early signs of diarrhea and dehydration, improving access to safe drinking water, providing children with proper and balanced nutrition, especially during illness, and increasing access to health centers to provide urgent services to children with diarrhea can be effective in reducing child hospitalizations.

As the results showed, it can also be claimed that most people who suffer from bloody diarrhea in this study are inpatients, and the number of inpatients is significantly higher than outpatients. To address the issue of generalizability, it is important to consider the specific characteristics of our study population, which may differ in their demographic and health status compared to those in other regions or healthcare settings. For instance, variations in healthcare delivery, access, and patients’ socio‐economic backgrounds could result in different distributions of the clinical status profile, thus affecting the applicability of our findings to other contexts. While our results provide valuable insights within the studied population, we recommend cautious interpretation when extrapolating these findings to broader populations, especially those with different healthcare systems or epidemiological profiles. Future studies should aim to explore these factors across diverse settings to improve generalizability and validate our findings.

In line with our study, various studies have shown that the *Shigella* pathogen continues to carry a significant cause of bloody diarrhea worldwide, particularly in Asia and Africa. Moreover, it is a highly virulent pathogen [2, 22–24]. However, it is important to recognize that other bacterial, viral, and protozoal pathogens also contribute to the disease burden, and in some cases, no pathogen is identified due to diagnostic limitations. Although *Shigella* serotypes and antimicrobial resistance patterns were not evaluated in the present study, previous studies provide important contextual evidence. A study conducted between 1995 and 2002 reported that *S. sonnei* was the most prevalent species (71.5%), followed by *S. flexneri* (22.6%) and *S. boydii* (1.8%), whereas no cases of *S. dysenteriae* were identified during that period. The shift toward S. sonnei as the predominant species contrasts with earlier data from the same region, where *S. flexneri* had been more common [25]. These findings suggest that the distribution of *Shigella* species may change over time, possibly due to improvements in sanitation, population immunity, or selective pressure from antibiotic use. *Shigella* overall accounted for more than half of the isolates, indicating that it continues to play a major role in the epidemiology of bloody diarrhea. Accurate and timely pathogen identification is critical not only for appropriate clinical management but also for guiding antimicrobial therapy. Misuse or overuse of antibiotics can drive resistance, complicating treatment and increasing healthcare costs [25]. In this case, the need to improve health infrastructure and promote health education in communities is considered a key strategy to reduce the burden of the disease and prevent its spread. Antimicrobial resistance patterns reported in previous studies provide important background context for interpreting the burden of shigellosis, although such data were not available in the present study. The earlier study demonstrated a marked increase in resistance to trimethoprim‐sulfamethoxazole (from 39% to 70%) and the emergence of multidrug‐resistant strains, particularly among S. flexneri. In contrast, no resistance to ciprofloxacin was observed during that period [25]. These findings highlight the potential public health importance of antimicrobial resistance in similar settings and underscore the need for local resistance data. While our study showed a decreasing trend in overall incidence, the persistence of resistant strains could pose a major challenge for long‐term disease control, highlighting the need to regularly monitor resistance and to use antibiotics more carefully to keep treatments effective. To effectively combat the *Shigella* pathogen, it is essential to invest in the research and development of new treatments and vaccines, which can help improve the public health situation in affected areas [26, 27].

Environmental factors, including temperature, humidity, and rainfall, may influence the epidemiology of bloody diarrhea. A study in Kilifi and Nairobi Counties demonstrated that there may be a positive correlation between acute bloody diarrhea and the monthly mean rainfall [24]. Additionally, research conducted in Iran showed that an increase in the number of rainy days per month is associated with an increased risk of bloody diarrhea in males [28]. Therefore, some infections can have seasonal patterns influenced by environmental factors such as temperature and moisture levels.

In our study, the highest number of cases of bloody diarrhea were observed in June and May, which various studies have shown that the incidence of bloody diarrhea and other intestinal infections is related to climate changes such as temperature and rainfall. The incidence of these diseases usually increases in months when temperature and humidity are higher (such as June and May) [24, 29]. Increased temperature and humidity increase the growth and survival of enteric pathogens such as *Shigella* in water and food, increasing the likelihood of contamination of water and food sources [24, 30]. Also, in warmer seasons, the likelihood of consuming contaminated water and food may be higher, and personal hygiene practices such as handwashing may also be less likely [24]. Increased agricultural activities and the use of surface water for irrigation during hot seasons, which may cause the transmission of pathogens to humans, and more social gatherings and activities during these seasons, can also contribute to the occurrence of bloody diarrhea [24, 31].

The data analysis showed a consistent decline in cases of bloody diarrhea over the years, which is likely to reach a stable level in the coming decades. This trend may reflect improvements in the control of related infectious diseases, advances in sanitation and healthcare, and increased public awareness of preventive measures. Also, given the development of diagnostic and therapeutic technologies, as well as better access to healthcare, this decline could continue. However, it should be noted that factors such as antibiotic resistance, climate change, and regional sanitation conditions may pose challenges in the long term that may affect the decline, as a review in Iran has shown that *Shigella* is showing an increasing trend [32]. Alternatively, it is important to note that the study period coincided with the COVID‐19 pandemic, which may have influenced the observed trends. During 2020–2022, public health restrictions, changes in healthcare‐seeking behaviors, and disruptions to routine surveillance systems could have led to underreporting of diarrheal cases [33]. On the other hand, better hygiene and preventive measures, such as handwashing, may have helped reduce cases and improved handwashing and reduced interpersonal contact, which may have contributed to a real decline in incidence. These factors should be considered when interpreting the decreasing trend observed in our study, as part of the reduction may be attributable to pandemic‐related changes rather than long‐term epidemiological patterns alone. Overall, the gradual decline, although promising, highlights the need for continued monitoring and preventive measures.

## 4. Limitation

We acknowledge several limitations of our study. First, this study relied on routine surveillance reporting systems, which are subject to underreporting and reporting bias. Mild cases of bloody diarrhea that did not seek medical care, particularly during periods of healthcare system strain such as the COVID‐19 pandemic, may not have been captured. Additionally, changes in healthcare‐seeking behavior, diagnostic capacity, and reporting practices over time could have influenced the observed trends. Second, diagnostic limitations should be considered. Laboratory confirmation depended on the availability of diagnostic methods, which may not detect all causative pathogens, particularly viral or protozoal agents, and may result in misclassification or underestimation of non‐*Shigella* etiologies. Furthermore, data on *Shigella* serotypes and antimicrobial resistance patterns were not available, limiting our ability to assess strain distribution and resistance trends in the study population. Third, the time‐series analysis using ARIMA models is based on several assumptions, including stationarity and consistent temporal patterns. Although model diagnostics suggested an acceptable fit, forecasting accuracy may be affected by unmeasured factors such as climate change, population dynamics, public health interventions, and future changes in surveillance or healthcare systems. Finally, the findings from Kerman Province may not be fully generalizable to other regions due to differences in environmental conditions, healthcare infrastructure, and population characteristics.

## 5. Conclusion

In this study, diverse factors were investigated between demographic and meteorological variables and the risk of bloody diarrhea, which may be influenced by several factors in different regions. Bloody diarrhea poses a significant health risk, particularly among young children, due to its potential to lead to severe dehydration and complications. Preventive strategies such as good hygiene, ensuring access to safe water, and promoting balanced nutrition are crucial. Furthermore, addressing socioeconomic determinants of health, such as access to healthcare and sanitation, is necessary for reducing the burden of diarrheal diseases globally. Continuous surveillance and investment in research and development of new treatments and vaccines are essential for combating these infections effectively. Overall, a multifaceted approach that combines improved preventive infrastructure, education, and healthcare is necessary to reduce the impact of bloody diarrhea and similar conditions.

## Funding

This research received no specific grant from any funding agency, commercial or not‐for‐profit sectors.

## Ethics Statement

The current study was carried out in accordance with the Declaration of Helsinki and was approved by the Ethical Committee of Shiraz University of Medical Sciences (Ethics Code: IR.SUMS.REC.1402.482).

## Conflicts of Interest

The authors declare no conflicts of interest.

## Data Availability

The data that support the findings of this study are available on request from the corresponding author. The data are not publicly available due to privacy or ethical restrictions.
